# Ptf1a is expressed transiently in all types of amacrine cells in the embryonic zebrafish retina

**DOI:** 10.1186/1749-8104-4-34

**Published:** 2009-09-04

**Authors:** Patricia R Jusuf, William A Harris

**Affiliations:** 1 Department of Physiology, Development and Neuroscience, University of Cambridge, Downing Street, Cambridge CB2 3DY, UK

## Abstract

**Background:**

The vertebrate retina is composed of five major types of neurons: three excitatory (photoreceptors, bipolar cells and ganglion cells) and two inhibitory (horizontal and amacrine cells). The transcription factor Ptf1a (pancreas transcription factor 1a) is important for the normal development of the inhibitory retinal neurons.

**Results:**

Using a transgenic Ptf1a:GFP reporter and *in situ *hybridization in the zebrafish retina, we show that *ptf1a *message is transiently expressed in all amacrine and horizontal cells within hours after the terminal division of multipotent progenitors at the apical surface of the retinal neuroepithelium, and remains on as these cells migrate to their final laminar location. The message then shuts off, but we can follow the stable Ptf1a:GFP protein for up to 120 hours post-fertilization. A variety of anatomically and neurochemically distinct subtypes of amacrine cells can already be distinguished at this embryonic time point.

**Conclusion:**

The timing of Ptf1a expression suggests that it is involved in the very early stages or steps in the differentiation of amacrine cells, which, due to the perdurance of the Ptf1a:GFP, can be seen to rapidly diversify into a large number of subtypes. This work sets the stage for future studies looking at genetic specification of amacrine subtypes.

## Background

The zebrafish has emerged as an important vertebrate model system of developmental studies due to its fast *in vitro *development, transparency, ease of molecular manipulations and the large variety of mutant and transgenic zebrafish strains generated. Ptf1a (pancreas transcription factor 1a) is a helix-loop-helix transcription factor that was first identified as a subunit of the trimeric PTF1 transcription factor complex [1], which is crucial for the development and maintenance of the pancreas [2-7]. Ptf1a was also shown to play an important role in the neurogenesis of different central nervous system structures. In particular, Ptf1a is important for the generation of many inhibitory (primarily γ-aminobutyric acid (GABA)-ergic) interneurons in different areas, such as the spinal cord [8-11] and cerebellum [7,12], although in specific other central nervous system regions it is also involved in the specification of excitatory glutamatergic neurons [13]. When Ptf1a is knocked down, the inhibitory cells that usually express Ptf1a during development become glutamatergic cell types [10,14].

In the retina, Ptf1a is expressed in the horizontal and amacrine cell populations. Studies in *Xenopus *and mouse retina show that Ptf1a is both essential and sufficient for determining the fates of these inhibitory neuronal types [15-17]. We made use of a transgenic zebrafish line expressing green fluorescent protein (GFP) under the control of the *ptf1a *promoter [5] to describe the expression of this gene in relation to the development of cells expressing Ptf1a. *In vivo *time-lapse movies in this line helped us determine that Ptf1a turns on in differentiating horizontal and amacrine cells within hours of the completion of the last progenitor division and stays on in these precursors until they begin to differentiate, at which point the Ptf1a transcript disappears. Ptf1a:GFP protein remains in these cells as they differentiate into a variety of subtypes.

Different types of photoreceptors and horizontal, bipolar and ganglion cells have already been described in detail in the adult zebrafish retina [18-21], but only a few anatomically distinct amacrine cell types have been noted [18]. Mosaic expression of the transgene combined with a morphometric classification scheme allowed us to distinguish 28 amacrine subtypes in the zebrafish retina at just 120 hours post-fertilization (hpf). This is the first comprehensive characterization of amacrine subtypes in the zebrafish retina, which will hopefully be useful in understanding visual function and the specification of subtype identity in this developmental model system.

## Results

### Transgenic Ptf1a:GFP expression marks cells expressing Ptf1a during development

To study the developing cell types that express Ptf1a during retinal neurogenesis, we made use of a transgenic line expressing GFP under the control of the *ptf1a *promoter (kindly provided by Professor Steven Leach) [5,22]. To ensure that the retinal cells that express Ptf1a:GFP reflect the expression of endogenous *ptf1a *gene, the spatio-temporal expression pattern of *ptf1a *RNA was studied using *in situ *hybridization and compared to the expression of GFP in the transgenic line.

**Figure 1:**
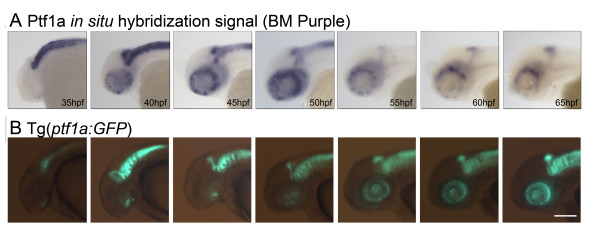
**Comparison of endogenous *ptf1a *RNA expression and GFP reporter protein**. Expression in wholemount transgenic zebrafish heads is shown at different ages (hours post-fertilization; times are identical in both panels). **(A) **Endogenous *ptf1a *RNA expression was revealed by *in situ *hybridization visualized by BM purple. **(B) **GFP reporter protein expression of Tg(*ptf1a:GFP*) embryos. Stable GFP expression follows endogenous RNA expression with a temporal delay of a few hours and is maintained. In contrast, endogenous RNA expression of *ptf1a *occurs relatively transiently and disappears from differentiated neural tissue by 65 hpf. At this age, it is only maintained in the ciliary margin of the eye, where neurons continue to develop throughout adulthood. Scale bar = 1 mm.

Endogenous *ptf1a *RNA was first expressed at 35 hpf in the ventronasal patch of the retina, as described for the *ath5 *gene, which drives ganglion cell fate [23]. Expression then spread nasally and dorsally, with the dorsal temporal region being the last to express *ptf1a *(Figure 1A). The *ptf1a *RNA expression was highly transient, being expressed at any given region for less than 5 hours. By about 60 to 65 hpf *ptf1a *is completely absent from all but the ciliary margin (where retinal development continues throughout adulthood). The onset of GFP expression in the Ptf1a:GFP transgenic line followed that of the RNA expression, with a delay of 4 to 5 hours, but accurately follows the pattern of *ptf1a *message expression - as one would expect of a good anatomical reporter. However, while RNA expression is very transient, the GFP protein is stable and is retained in the retinal cells throughout development (Figure 1).

### Ptf1a is transiently expressed in individual retinal cells as they leave the cell cycle and begin to differentiate

Many factors that bias progenitors to adopt a distinct fate are expressed prior to the terminal division of a progenitor cell. We have previously shown that the transcription factors Vsx1 and Ath5 turn on in progenitors just before they undergo a final division at the apical surface of the developing retina, thus restricting the fate choices of both daughters of such progenitors [24]. Ptf1a has previously been shown to be necessary in the differentiation of horizontal and amacrine cells in the retina [15-17]. Labelling with a 1 or 2 hour bromodeoxyuridine pulse suggested that Ptf1a turns on postmitotically in mouse retina [16,17]. We made use of the four-dimensional imaging capability in the zebrafish to directly address the question at which stage of the cell cycle/differentiation process is this gene switched on in individual cells.

Cells undergo their final (and all previous) cell division as multipotent progenitors (reviewed by [25]) at the apical surface and then migrate to their appropriate layer in the developing retina, taking 3 to 6 hours to reach their final laminar position (measured from Supplementary Movies 4 and 5 in [24]).

**Figure 2:**
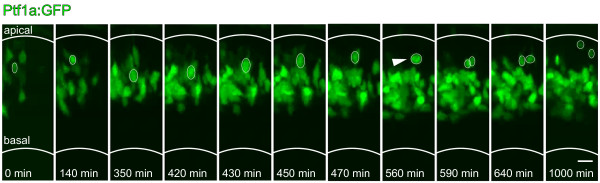
**Temporal onset of Ptf1a expression in Tg(*ptf1a:GFP*)**. GFP-positive cells migrate to the centre of the neuroepithelium, where they form a wide band that will become the amacrine cell layer. Some cells (one example marked with white oval) move from this band back apically to the horizontal cell layer, where they undergo an additional division (white arrowhead shows mitosis). No cells are seen to move back to the apical surface and divide as would be expected from progenitor cells. Scale bar = 10 μm.

Imaging of developing retinas from Tg(*ptf1a*:*GFP*) embryos from 30 hpf for 16 to 28 hours revealed that GFP first turns on in cells in the apical half of the neuroepithelium within the first few hours following their terminal neuroepithelial division. GFP positive cells then migrate basally until they reach the central part of the epithelium, the approximate laminar position of future amacrine cells. Some of these GFP positive cells then move back apically to the developing horizontal cell layer in a process that depends on Lim1 expression [26,27]. Here, they undergo one last division (Figure 2) to produce a pair of sibling horizontal cells and subsequently extend processes into the developing outer plexiform layer (reviewed by [28]). This remarkable non-ventricular division of differentiating horizontal cells has been described in detail by Godinho *et al*. [22].

**Figure 3:**
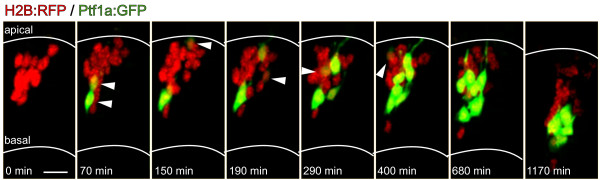
**Ptf1a turns on in postmitotic differentiating cells**. Time-lapse images from Additional file 1 (38 to 58 hpf) showing cells transplanted from a Tg(*ptf1a:GFP*)/*H2B:RFP *RNA-injected embryo to reveal individual cells. Some transplanted (red) cells turn on Ptf1a:GFP expression in the apical half (top) of the developing neuroepithelium, but not always at the apical surface. White arrowheads indicate cells that have turned on expression of Ptf1a:GFP in that frame. Location of cells indicated by white arrowheads reveal that Ptf1a:GFP in individual cells turns on at different stages of their migration (different depth between apical surface and developing amacrine layer in the middle of the neuroepithelium). Scale bar = 10 μm.

To study individual cells, time-lapse movies were made of wild-type embryos into which only a few cells from Tg(*ptf1a*:*GFP*) donors were transplanted. The entire population of transplanted cells was visualized by injection of *H2B:RFP *RNA into the yolk of the donor Tg(*ptf1a*:*GFP*), thus marking all transplanted nuclei with red fluorescent protein (RFP). In these transplanted embryos, many red nuclei are seen to undergo terminal division at the apical surface and then turn on Ptf1a:GFP expression subsequently (Additional file 1). These cells are at various stages of their migration (see position of cells as Ptf1a:GFP turns on; arrowheads in Figure 3), but never move up to the apical surface to undergo division again (Figure 3). It was not easy to follow individual cells backwards to their last division, but we can say that the last division of any H2B:RFP-positive nuclei was observed at least 160 minutes prior to Ptf1a:GFP expression.

This delay of hours and positioning and subsequent behaviour of cells turning on Ptf1a:GFP matches the 3- to 6-hour migration time observed previously in developing zebrafish retinal neurons (Supplementary Movies 4 and 5 in [24]) and the delay of approximately 5 hours in onset of GFP compared to the endogenous RNA expression reported here.

**Figure 4:**
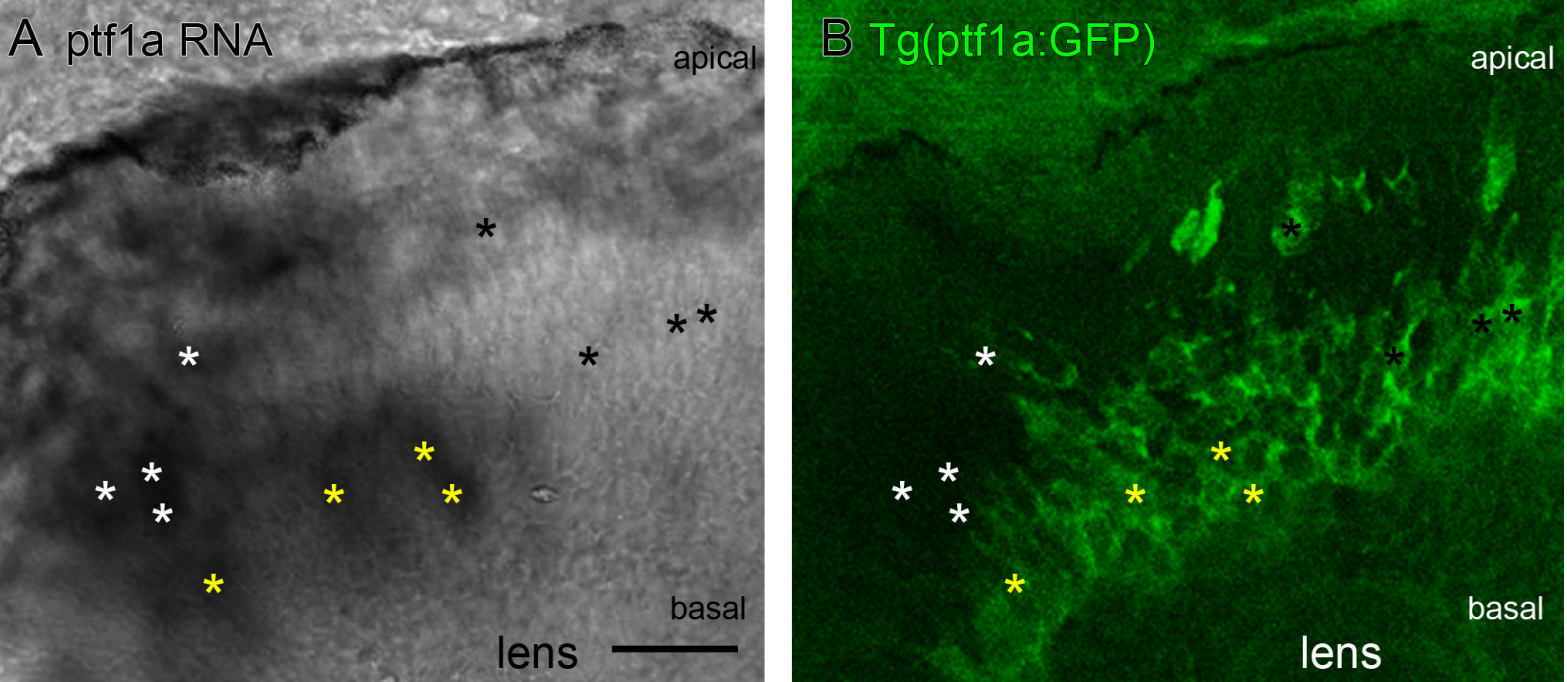
**The transition from *ptf1a *RNA to ptf1a:GFP protein expression**. Coronal section through Tg(*ptf1a:GFP*) embryos with *in situ *hybridization labelled endogenous *ptf1a *RNA expression at 48 hpf. **(A) **Endogenous *ptf1a *RNA expression as revealed by BM purple visualized *in situ *hybridization. Labelled cells are found near the apical surface and in the middle of the developing retinal neuroepithelium. **(B) **Tg(*ptf1a:GFP*) embryos show a large band of labelled cells in the centre of the neuroepithelium, where differentiating amacrine cells form part of the inner nuclear layer. A few labelled cells are found more apically, where horizontal cells make up the outermost layer of the inner nuclear layer. In some regions apical cells express only the endogenous ptf1a RNA, but not GFP (white asterisks), some co-express both (yellow asterisks), and cells already in the future amacrine layer primarily express only GFP (black asterisks). Scale bar = 20 μm.

Staining patterns consistent with the time-lapse data were observed in sections co-labelled for *ptf1a *RNA by *in situ *hybridization and Ptf1a:GFP reporter (Figure 4). Endogenous *ptf1a *RNA expression is seen in cells in the apical half of the retinal neuroepithelium (white asterisks in Figure 4 mark individual cells that show *in situ *signal but no Ptf1a:GFP expression). These might represent cells that have recently undergone terminal cell division at the apical surface. Ptf1a:GFP expression in these sections is found primarily in the middle of the neuroepithelium, where differentiating amacrine cells are found. Because the Ptf1a:GFP expression is maintained for longer than the transient RNA expression (Figure 1), it is not surprising to find that many of the Ptf1a:GFP cells that are located in the future amacrine cell layer do not express *ptf1a *RNA (black asterisks in Figure 4 indicate Ptf1a:GFP-positive, *in situ *signal-negative cells). We suggest that these cells are likely to represent the population that used to, but no longer, expresses *ptf1a *RNA. Overlap of the RNA *in situ *signal and GFP occurs, as expected if this suggestion is correct, in cells at the junction of these two regions (yellow asterisks in Figure 4 show double labelled cells). The most likely interpretation is thus that endogenous but transient *ptf1a *RNA expression turns on in cells apically around the time of the terminal division and is downregulated as cells migrate to their final positions, whilst the Ptf1a:GFP expression turns on in migrating cells a few hours after the RNA expression and is maintained as cells migrate to their final laminar destinations in the retina and differentiate.

Because the time of onset and finish of RNA expression in cells is very tightly regulated, we suppose that the Ptf1a transcription factor itself might also be highly transient and expressed only briefly during early amacrine and horizontal differentiation. However, without a suitable antibody in this species, we cannot know how long the native Ptf1a protein might act within these cells.

### Ptf1a:GFP is expressed in all inhibitory cells of the embryonic retina

**Figure 5:**
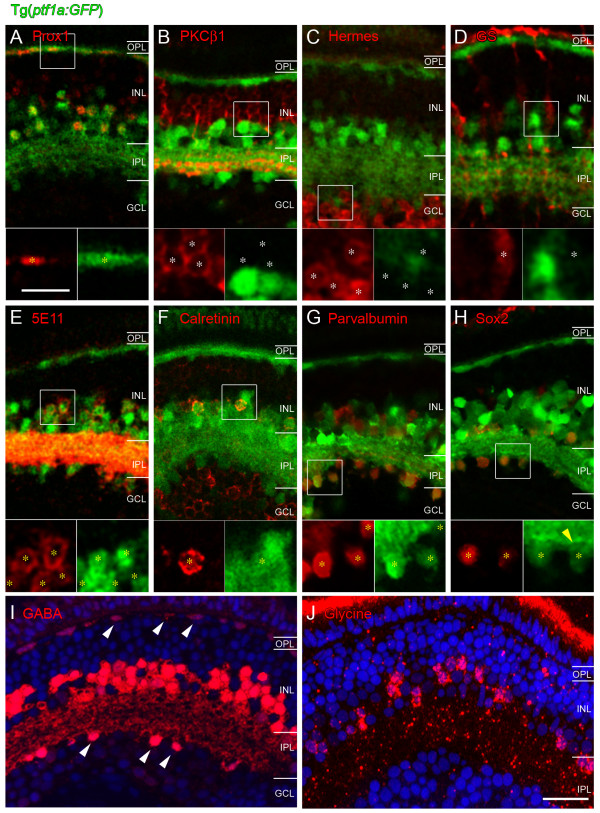
**Immunohistochemical characterization of GFP-expressing cells in Tg(*ptf1a:GFP*) embryos at 120 hpf**. Antibodies used are shown in each panel and appear in the red channel. Insets show higher magnification in which all red (antibody marker) cells are marked by asterisks. White asterisks show red cells that are Ptf1a:GFP negative (B-D), yellow asterisks show red cells that are Ptf1a:GFP positive (A, E-H). **(A) **All horizontal cells as identified by Prox1 immunoreactivity also express Ptf1a:GFP. Prox1 also weakly labels bipolar cells, which do not express Ptf1a:GFP and a subpopulation of amacrine cells, which does co-label with the Ptf1a:GFP. **(B-D) **Ptf1a:GFP expressing cells do not colocalise with the bipolar cell marker PKCβ1, the ganglion cell marker Hermes (including the displaced amacrine cells in the ganglion cell layer) nor the Müller cell-specific marker glutamine synthetase. **(E-H) **In contrast, Ptf1a:GFP-expressing amacrine cells colocalise with the pan-amacrine marker 5E11, and with the amacrine subpopulation markers calretinin, parvalbumin and Sox2. Calretinin (F) is also expressed in ganglion cells and does not label the displaced amacrine populations. **(I, J) **GABA and glycine staining in plastic sections reveal that some cells in the amacrine layer of the INL express GABA and some express glycine. Some cells in the outermost INL (horizontal cells) and ganglion cell layer (displaced amacrine cells) label with GABA (white arrowheads), but never glycine. The nuclear stain DAPI was used to label the retinal layers. GABA, γ-aminobutyric acid; GCL, ganglion cell layer; GS, glutamine synthetase; INL, inner nuclear layer; IPL, inner plexiform layer; OPL, outer plexiform layer; PKC, protein kinase C. Scale bar in (J) = 20 μm and applies to (A-J); scale bar in the inset of (A) = 10 μm and applies to insets in (A-H).

To establish which neural types express Ptf1a, sections through Tg(*ptf1a*:*GFP*) embryos were labelled immunohistochemically with neuron type- or subtype-specific markers. As expected from previous studies in other vertebrates, Ptf1a-expressing cells in the zebrafish were not co-labelled with markers for the glutamatergic photoreceptors (Zpr1 and Zpr3; data not shown), bipolar cells (protein kinase C (PKC), Figure 5B), ganglion cells (Hermes, Figure 5C) or glial Müller cells (glutamine synthetase, Figure 5D; white asterisks in Figure 5B-D show cells labelled red by antibodies that are not co-labelled with Ptf1a:GFP).

Ptf1a:GFP cells did, however, co-label with the pan horizontal (Prox1, Figure 5A) and pan-amacrine (5E11, Figure 5E; yellow asterisks in Figure 5A,E show cells double labelled for antibody staining and Ptf1a:GFP) markers. A variety of subtype-specific markers of amacrine cells were used to see if distinct populations are already present at this age. These included calretinin (which additionally labels ganglion cells in the zebrafish retina; Figure 5F), parvalbumin (Figure 5G) and Sox2 (Figure 5H). All of these co-labelled with Ptf1a:GFP-expressing amacrine cells in the inner nuclear layer (INL) and some co-labelled with displaced amacrine cells in the ganglion cell layer (yellow asterisks in Figure 5F-H show that cells labelled by antibodies are also positive for Ptf1a:GFP). Crisp staining for GABA or glycine could only be achieved in plastic sections (in which the GFP of the Ptf1a:GFP could not be recovered), and these revealed that about half of the cells in the amacrine layer (inner half of the INL) are GABA immunopositive and about a third are glycine immunopositive (Figure 5I, J). Horizontal (elongated somas in the outermost layer of the INL) and displaced amacrine cells (ganglion cell layer (GCL)) seem to be exclusively GABAergic (white arrowheads in Figure 5I), as no glycine staining was observed in the outer INL or GCL (Figure 5J).

We conclude that Ptf1a is not expressed in the excitatory cell types, (photoreceptors, bipolars and ganglion cells) or in Müller cells, but is exclusively expressed in inhibitory neurons (horizontal and amacrine cells). We also conclude that immunohistochemically distinct subtypes of Ptf1a-expressing cells are already established at 120 hpf.

### Morphological classification of Ptf1a-expressing amacrine cell subtypes

Since the immunohistochemical labelling revealed the existence of specific subtypes, we wondered if the large variety of amacrine subtypes found in adult retinas is already established at this early developmental stage and, if so, how many different subtypes are found in the zebrafish. We therefore undertook a morphological classification of the different types of Ptf1a-expressing cells present at this early developmental stage. As *ptf1a *RNA is expressed transiently, there is a limited pool of Ptf1a:GFP in each precursor cell. Thus, continued frequent imaging of Ptf1a:GFP-expressing cells led to the bleaching of the GFP signal, so we decided to survey these cells at 120 hpf. Furthermore, Godinho *et al*. [29] previously studied the early steps of neurite outgrowth in amacrine cells using time-lapse series in different transgenic lines. Amacrine cells migrate to the developing INL and sprout processes in random directions. Those processes that are directed towards the ganglion cells are stabilized and arborise laterally in the forming inner plexiform layer (IPL) in either 'on' or 'off' sublaminas, suggesting that initial stages of amacrine subtype specificity are already initiated by this stage [29].

Prox1 labelling revealed that Ptf1a:GFP is expressed in all horizontal cells and whilst different morphologies of Ptf1a:GFP-expressing horizontal cells were encountered in this study, these have recently already been characterized in great detail [21]. Our sample allowed for a vertical view of cells only (not a wholemount view) and horizontal cells with different sized somas with and without axons were seen (Additional file 2), but without the more informative wholemount view revealing patterns of dendritic tips, these cells could not be effectively classified here.

Since Ptf1a:GFP labels the entire inner half of the INL (amacrine cell layer), we could use it to determine the subtypes of amacrine cells (the only type of retinal neuron in the zebrafish whose subtype variety has not yet been described extensively) that arise from Ptf1a-expressing progenitors at 120 hpf. At least 25 to 30 amacrine cells have been described in other adult vertebrate retinas [30-32] and the large variety reflects the important function these interneurons play in the different parallel visual circuits in the retina. A few morphological amacrine cell types have been described previously in the adult zebrafish retina [18], and we asked if these and possibly additional types would be found in the 120-hpf zebrafish retinas. Previous data show that neuritic outgrowth of amacrine cells stabilises by 70 to 73 hpf and lamination and mature synapses are also evident by this stage [29,33,34]. Similarly, visual, electroretinogram and optokinetic reflex responses can be recorded around this time [33,35,36]. This suggests that subtypes of cells have established appropriate connectivity even by 3 or 4 days post-fertilization, such that functionally different subtypes are probably apparent by 120 hpf.

**Figure 6:**
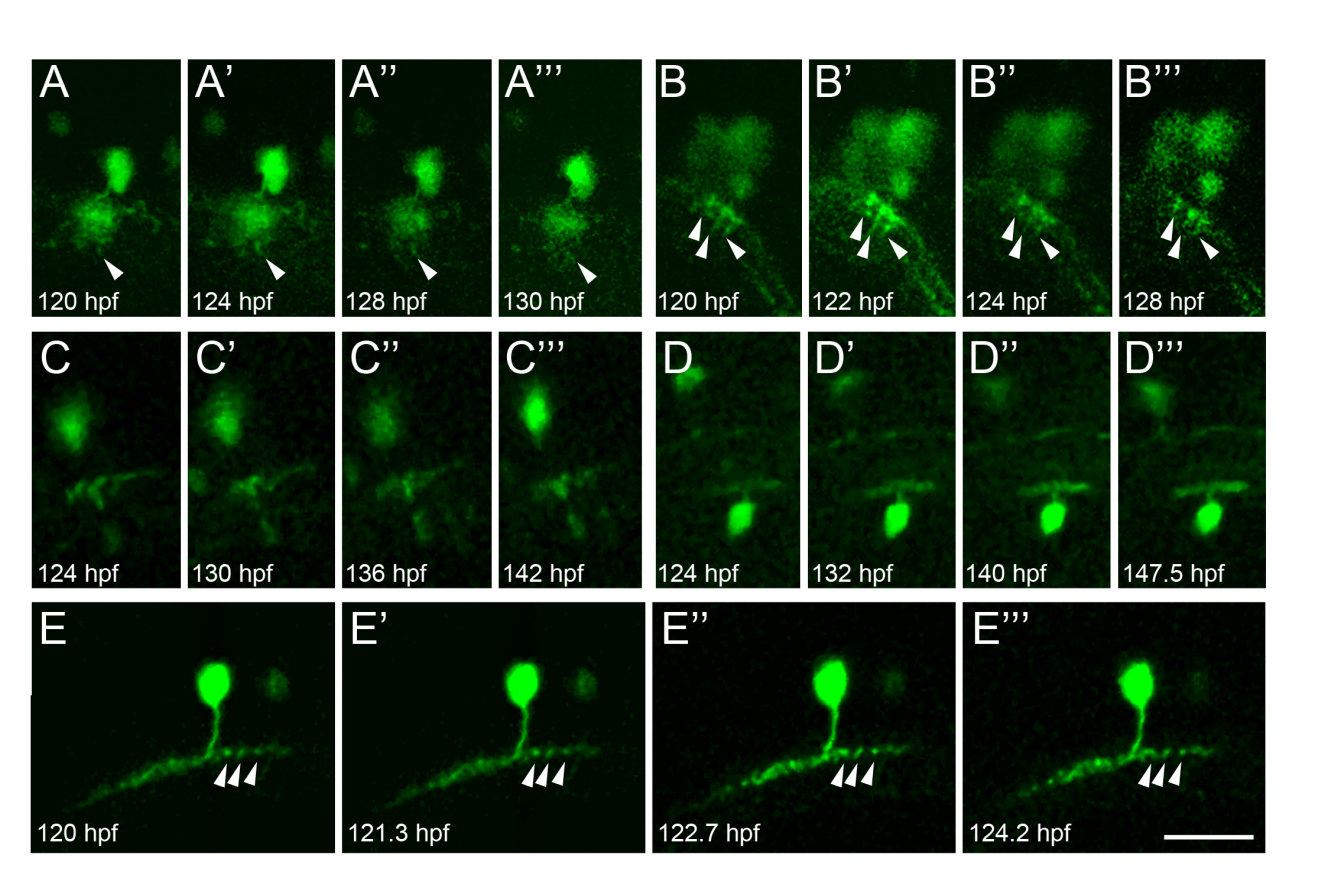
**Ptf1a:GFP amacrine cells at 120 hpf have stable neurite arbors**. Single time-lapse images of different amacrine cells imaged for up to 24 hours. The GFP signal is relatively weak in some cells but, nonetheless, the stratification depth extent and lateral neurite arbor width remain unchanged over the imaging period. In some brighter labelled cells, even individual branches or varicosities are seen to remain stable (white arrowheads), in contrast to the rapid neurite remodelling of amacrine cells, which has been previously described up till 73 hpf [29]. Scale bar = 20 μm.

Time-lapse analyses of 120-hpf amacrine cells were performed to see how stable neuritic arbors are at this stage. Individual cells were obtained by injecting *ptf1a*:*GFP *DNA construct into the cap cells of a one- or two-cell stage embryo, which results in a mosaic expression pattern. Stacks of images were taken through retinas of live wholemount embryos at 120 hpf. The cells shown were extremely stable, and some of the detailed branches seemed to remain unchanged for up to 24 hours (white arrowheads in Figure 6), in contrast to the highly dynamic branches around 60 hpf, which have been shown previously to change within 20 minutes [29].

**Figure 7:**
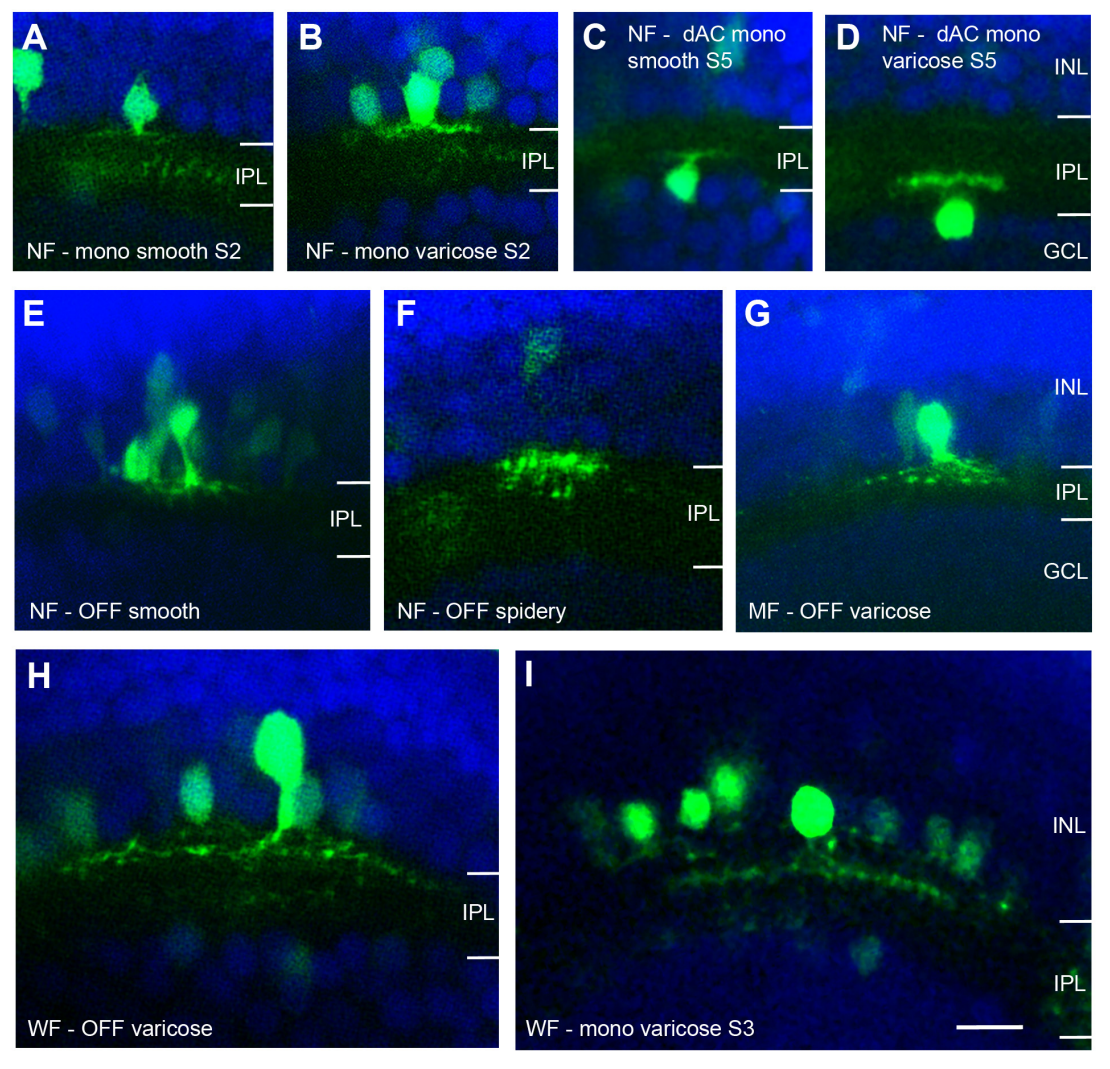
**Morphology of different types of amacrine cells in Ptf1a:GFP DNA-injected embryos (120 hpf)**. Micrographs show single images or extended focus views to reveal most of the morphology of individual cells without interfering neurites from neighbouring labelled cells. The nuclear stain DAPI was used to reveal the retinal layers. Their somas are found in the inner half of the INL or in the GCL immediately adjacent to the IPL. Their neurites can be smooth or beaded (with varicosities) and differ in width, stratification depth, and stratification breadth. **(A-F) **Narrow-field (NF) amacrine cell type examples that are monostratified (A, B), displaced (somas in the GCL in (C, D)) and monostratified, or multistratified (E, F). **(G) **Medium-field (MF) amacrine cell with varicose processes stratifying throughout the OFF sublamina. **(H, I) **Examples of two wide-field (WF) amacrine cells stratifying broadly in the OFF sublamina (H) or narrowly in stratum 3 (I). dAC, displaced amacrine cell; GCL, ganglion cell layer; INL, inner nuclear layer; IPL, inner plexiform layer; mono, monostratified; S, stratum. Scale bar = 10 μm.

**Figure 8:**
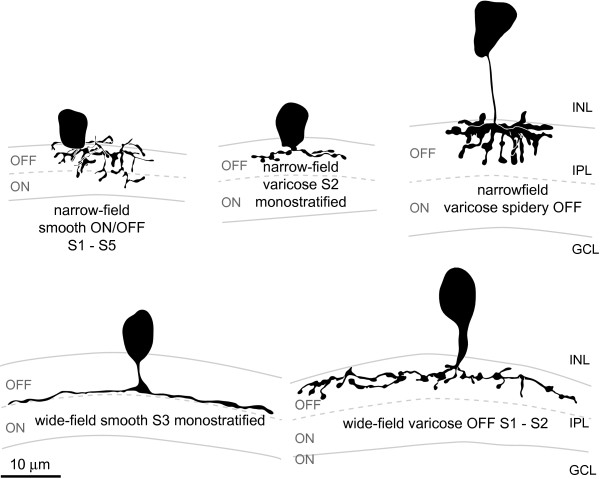
**Amacrine cell types**. Drawings of amacrine cell types showing the detailed morphology of their processes and their stratification within the inner plexiform layer (OFF and ON sublamina border indicated by dashed line). Cells are labelled according to the descriptive nomenclature used in Table 1, which summarises the depth and breadth of stratification, relative size of neurite arbor width and general morphology of processes. GCL, ganglion cell layer; INL, inner nuclear layer; IPL, inner plexiform layer. Scale bar = 10 μm.

**Figure 9:**
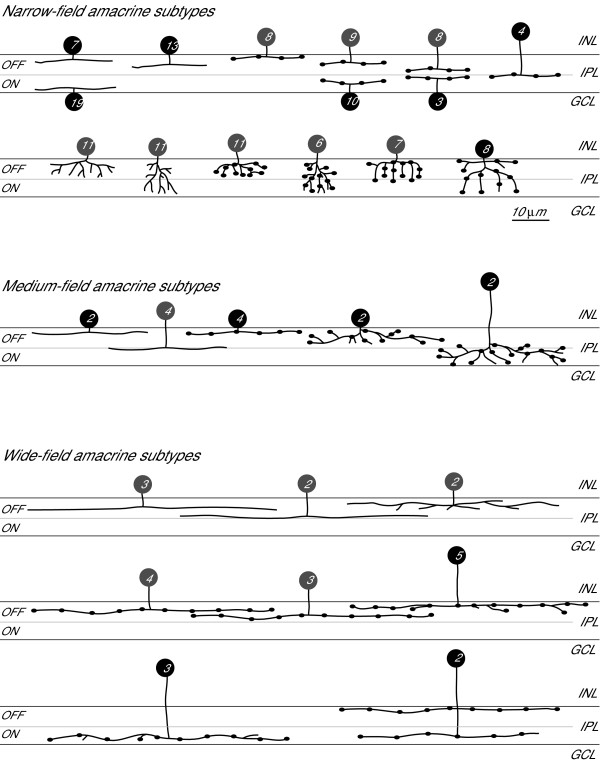
**Schematic drawings of the 28 different types of amacrine cells encountered**. Smooth or varicose narrow-field amacrine cells (15 types) were mono- (9 types) or multistratified (6 types). The three displaced amacrine cell types (somas in the GCL) found were all monostratified within the ON sublamina. Among the multistratified types, two stood out not only by their stratification depth, but also because they had the morphology of 'spidery' amacrine cells described in mammals [37]. Five types of medium-field amacrine cells and eight types of wide-field amacrine cells were found, which also came in smooth and varicose varieties and all showed characteristic stratification (mono- versus multistratified and depth of stratification). The location of black somas indicate where the majority of cells within this subtype had their somas (closest to the IPL, closest to the bipolar/outer half of the INL or inbetween these two layers). The locations of somas of subtypes shown in grey were randomly distributed throughout the inner nuclear layer. GCL, ganglion cell layer; INL, inner nuclear layer; IPL, inner plexiform layer. Scale bar = 10 μm.

Classification was performed on stacks of images taken through fixed wholemount embryos at 120 hpf. Micrographs of different morphologies of amacrine cells encountered are shown in Figure 7 (and more types are shown in Additional file 3) and traces of some example cells are shown in Figure 8. A total of 175 cells were analysed from stacks of images through the zebrafish eye (comparable to a vertically sectioned view of the retina) according to the size and location of their somas and morphology of their neurite arbors: width, stratification depth in the IPL, stratification breadth and distinguishing morphology (varicosities) of branches or shapes of processes (Table 1, Figure 9). Measurements were done by going through individual focal levels (not projections) using ImageJ and cells were included in these analyses only if they were spatially completely separated in three dimensions (that is, either in X, Y or focal depth) from surrounding labelled cells.

**Table 1:** Morphometric analysis of zebrafish amacrine cell subtypes

	**Soma location**	**Neurite arbor width mean ± SD (μm)**	**Stratification type**	**Neurite stratification depth in the IPL**	**Number of cells**
Narrow-field amacrine cell type					
Smooth 1	1	16.59 ± 5.53	Monostratified	S1	7
Smooth 2	1	16.48 ± 5.46	Monostratified	S2	13
Smooth OFF	1, 2, 3	14.22 ± 5.07	Broadly stratified	S1 to S2/3	11
Smooth ON/OFF	1, 2, 3	11.26 ± 4.93	Broadly stratified	S1/2 to S5/6	11
Varicose 1 *	1, 2, 3	14.32 ± 3.77	Monostratified	S1	8
Varicose 2	1, 2	19.44 ± 5.27	Monostratified	S2	9
Varicose 3	1, 2, 3	21.98 ± 7.58	Monostratified	S3	8
Varicose 4	2	23.47 ± 7.49	Monostratified	S4	4
Varicose OFF	1, 2	16.96 ± 3.84	Broadly stratified	S1 to S3	11
Varicose ON/OFF	1, 2	13.85 ± 6.80	Broadly stratified	S1/2 to S5/6	6
Varicose spidery OFF	1, 3	15.55 ± 4.45	Broadly stratified	S1 to S3/4	7
Varicose spidery ON/OFF	1	11.65 ± 8.44	Broadly stratified	S1 to S6	8
Displaced smooth 6	GCL	14.46 ± 5.02	Monostratified	S5/6	19
Displaced varicose 4	GCL	14.84 ± 2.40	Monostratified	S4	3
Displaced varicose 5	GCL	15.98 ± 6.64	Monostratified	S5/6	10
					
Total narrow-field cells					135
					
Medium-field amacrine cell type					
Smooth 1	1	36.98 ± 1.4	Monostratified	S1	2
Smooth 3/4	1, 2	29.02 ± 7.62	Monostratified	S3/4	4
Varicose 1	1	31.89 ± 3.84	Monostratified	S1	4
Varicose OFF *	1	30.99 ± 4.40	Broadly stratified	S1 to S3	2
Varicose ON	3	33.41 ± 8.37	Broadly stratified	S3 to S6	2
					
Total medium-field cells					14
					
Wide-field amacrine cell type					
Smooth 2	1, 3	63.0 ± 15.77	Monostratified	S2	3
Smooth 3/4	1, 2	57.19 ± 2.63	Monostratified	S3/4	2
Smooth OFF	1, 3	46.98 ± 11.94	Broadly stratified	S1 to S2/3	2
Varicose 2	2, 3	55.38 ± 13.74	Monostratified	S2	4
Varicose 3	1, 3	43.72 ± 2.79	Monostratified	S3/4	3
Varicose OFF *	3	49.81 ± 10.70	Broadly stratified	S1/2	5
Varicose ON *	3	65.09 ± 18.96	Broadly stratified	S5/6	3
Varicose ON/OFF bistratified	3	79.12 ± 47.4	Bistratified	S1/2 and S5/6	2
					
Total wide-field cells					24
					
Total number of classified cells					173
Number of unclassified cells *					2

Soma location: 1, innermost amacrine cell layer; 2, middle amacrine cell layer; 3, outermost amacrine cell layer. *Subtypes described by Connaughton *et al*. [18]: NF varicose 1 (Aoff-s1n); NF smooth ON/OFF (Adiffuse-1); MF varicose OFF (Aoff-s2); WF varicose OFF (Aoff-s1w); WF varicose ON (Aon-s5); WF varicose ON/OFF bistratified (Aon-s1/s5); unclassified (Aon-s4). GCL, ganglion cell layer; MF, medium-field; NF, narrow-field; S, stratum; SD, standard deviation; WF, wide-field.

The IPL was subdivided into six equal sublamina (S1 to S6) and cells were classified as monostratified (processes in one stratum only) or multistratified (all strata containing any processes were considered) in the outer (OFF), inner (ON) or both sublaminas. Amacrine cells in mammals have been further subdivided into narrow-, medium- and wide-field classes according to the width (presumed equivalent to the diameter) of the neurite arbors. Cells with large neurite arbors sample across large areas of the visual field and can thus modulate circuitry at different locations, which might be important, for example, for directionally selective behaviour, whereas narrow-field amacrine cells often provide modulation between different circuits (at different stratification levels) within the same receptive field and can thus function in linking OFF and ON circuits, as is the case for the AII amacrine cell in the rod pathway.

In some species, the width of neuritic arbors of amacrine cells naturally form separate groups, supporting the notion that cell sizes are not random [37]. Because of the much smaller size and difference in morphology of amacrine cells in the zebrafish at this age (120 hpf), the parameters could not simply be rescaled (by normalising to IPL depth or visual angle). Instead, our sample set was analysed for naturally occurring cutoffs in neuritic arbor width. Thus, cells were first grouped based on stratification depth, stratification breadth and morphological features of the processes (branching pattern, varicose or smooth). Within each of these groups, individual cells were ranked by neurite width and we looked for large breaks within the sizes to see if cells were part of a continuum of sizes or were perhaps multiple types. Such breaks (about 10 μm) were indeed observed at cell widths of 25 and 40 μm. Thus, narrow-field cells were defined as cells with arbor widths of up to 25 μm, medium-field cells as cells with arbor widths between 25 and 40 μm and wide-field cells as cells with arbor widths larger than 40 μm. Some types with the same stratification pattern fell into two of the categories by size, whereas others only occurred in one of these size-defined categories. Whilst this provides some confidence in the classification scheme presented here, we emphasise that without physiological studies, we cannot confirm any functional meaning behind these subdivisions.

Narrow-field amacrine cell types (15 types) were found relatively frequently (78% of all cells; Table 1) and came in mono- or multi-stratified varieties with distinct neurite morphologies (Figure 9). Many of the subtypes had somas that were located in the INL layer closest to the IPL, but other types had somas with variable positions within the depth of the INL. The arbors of medium-field (five types) and wide-field (eight types) amacrine cells were much simpler (Figure 9), being primarily mono-stratified (or multi-stratified within the same half of the IPL). Larger cells tended to have their somas located in the centre of the INL closest to the bipolar cell layers.

Neurites of most amacrine cells with somas in the INL stratified in the outer half of the IPL, whereas most of the neurites from displaced amacrine cells with somas in the ganglion cell layer stratified in the inner half of the IPL (Figure 9). Whilst displaced amacrine cells generally follow this trend in other species, INL amacrine cells often stratify throughout both sublaminas (reviewed in [38]).

Because some immunohistochemical markers for subtypes of amacrine cells seemed to label somas within a particular layer in the INL, the position of the soma (either closest to the IPL, closest to the outer bipolar half of the INL or in-between) was also noted. Some types had very characteristic locations of somas and are indicated by black somas in Figure 9, whilst other types had somas in various layers (grey somas in Figure 9), which suggests either that different types were grouped together or that soma position is not a cell type-specific feature. Soma size varied within subtypes and did not show greater variance between groups. On average, zebrafish amacrine somas at 120 hpf have a minor diameter (generally the width of the soma) of 5.14 μm and a major diameter (length of soma) of 7.1 μm.

**Figure 10:**
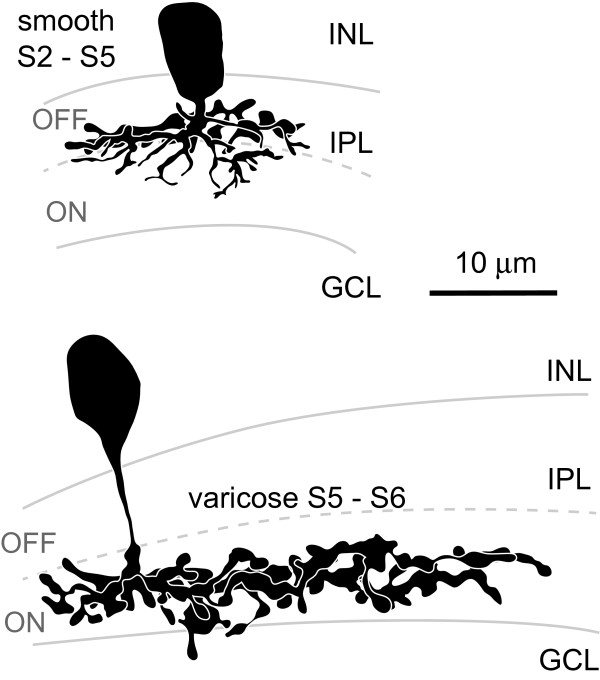
**Unclassified amacrine cells**. One cell is multistratified in the middle of the IPL, extending to OFF and ON sublaminas (border indicated by dashed lines) but not as close to the INL or GCL as other multistratified types do. The other is a wide-field cell that stratifies in S5/S6, but is branched much more densely than any other wide-field cell encountered with this stratification depth. GCL, ganglion cell layer; INL, inner nuclear layer; IPL, inner plexiform layer. Scale bar = 10 μm.

In sum, 28 different subtypes of amacrine cells were discovered (Table 1, Figure 9). Of these, 25 had somas in the INL and 3 types had somas displaced to the GCL. Narrow-field cells came in 15 subtypes, including all 3 displaced amacrine cells; 5 subtypes were medium-field, and 8 subtypes were wide-field. Additionally, two cells were found with morphologies that did not fit into any of these 28 subtypes (Figure 10). The fact that these isolated examples were found (which were not included in the classified types, each of which is represented by two or more examples) suggests that rarer subtypes might have been missed in this sample. We used a binomial statistics test that predicts that, at the 5% confidence level, our sample probably contains all types that occur at a frequency greater than 1.7%, but that rarer types might have been missed.

Connaughton and colleagues [18] described seven subtypes in adult zebrafish, and examples of each of these were found in our sample (including one of the isolated examples found in this study (Table 1), which indicates the equivalent types between the two studies). The actual sizes of our individual cells were smaller than those in the adult, although the relative size (compared to the size of the IPL and retina) were equivalent, suggesting that individual cells in the zebrafish retina continue to grow whilst retaining their distinct morphological features.

## Discussion

**Figure 11:**
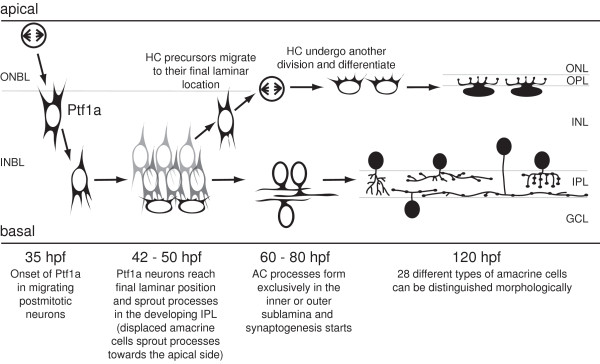
**The developmental progression of Ptf1a-expressing horizontal and amacrine cells in the retina**. Results used to draw this schematic come from this study and [29,33]. Beginning at 35 hpf, some multipotent progenitors undergo their terminal division and migrate towards their final laminar position (unconstrained mode) over the next 3 to 6 hours. During this migration, Ptf1a:GFP expression is turned on. Once settled at their correct position, processes start extending towards the developing IPL (basally for amacrine cells with somas in the INL, apically for amacrine cells that will be displaced to the ganglion cell layer). Some of these Ptf1a:GFP-expressing cells migrate back apically, reach their final layer, undergo a last division as committed horizontal progenitors and subsequently differentiate as horizontal cells. Processes of differentiating amacrine cells in the INL start extending laterally in the IPL either in the outer or inner half. By 120 hpf, horizontal and amacrine cell types have differentiated and 28 different subtypes of amacrine cells can be distinguished. AC, amacrine cell; GCL, ganglion cell layer; HC, horizontal cell; hpf, hours postfertilization; INBL, inner neuroblastic layer; INL, inner nuclear layer; IPL, inner plexiform layer; ONBL, outer neuroblastic layer; ONL, outer nuclear layer; OPL, outer plexiform layer.

Our study provides a detailed description of the dynamic expression of Ptf1a in zebrafish horizontal and amacrine cells. The *ptf1a *RNA is turned on very transiently in cells around or just after the time of their final apical mitosis, but is downregulated as these cells migrate to their final laminar position. Based on the Ptf1a:GFP reporter expression, the Ptf1a protein is probably expressed hours after the terminal division, as cells migrate. *Ptf1a *RNA expression turns off as or before migrating cells reach the target layer and new Ptf1a protein can thus no longer be made in these differentiating cells. We confirm the inhibitory cell fate of Ptf1a-expressing precursors as they account for the great majority, if not all, of the amacrine and horizontal cells and no other neuronal or glial cell type in the retina. Interestingly, subtype-specific markers are already expressed in 120-hpf embryos. That these neurons immediately differentiated into specific subtypes is further confirmed by our morphometric analysis of 28 amacrine cell subtypes in the zebrafish. Our results are combined with previous studies to summarise the developmental stages of Ptf1a-expressing retinal neurons (Figure 11) [29,33].

Ptf1a:GFP is expressed after the last division of multipotent progenitor cells (of pseudostratified undifferentiated morphology), although committed horizontal cell precursors (but not amacrine cells) do undergo an additional division in the outer neuroblastic layer [22]. These data are consistent with those in mouse showing that no Ptf1a-expressing cells incorporated bromodeoxyuridine during a 1- or 2-hour pulse prior to sacrifice [16,17]. Some examples that we were able to follow in four-dimensional movies show that Ptf1a:GFP turns on hours after the last division in cells between the apical side (where terminal mitosis occurs) and the centre of the neuroepithelium (where the future amacrine cell layer is located). The delay of up to 5 hours between the beginning of the RNA *in situ *signal and Ptf1a:GFP onset matches the delay of hours between individual cells expressing GFP after terminal division, as judged by the position of cells (along the apical-basal dimension) and subsequent migration of cells that turn on GFP, combined with the 3 to 6 hours between division and migration to the final layer observed previously [24]. These findings suggest that transcription of *ptf1a *is initiated around the time of terminal mitosis, but the protein and its effect on other genes as a transcription factor occurs during migration to the correct laminar position.

Ptf1a:GFP is exclusively expressed in the two inhibitory neuron types (horizontal and amacrine cells), but not in any of the excitatory neuron types of the retina, nor the glial cells, consistent with reports in other species [15-17]. Interestingly, amacrine subtype-specific markers (calretinin, parvalbumin and Sox2) also co-labelled Ptf1a:GFP cells, suggesting that different subtypes of amacrine cells arising from the Ptf1a lineage are already established at this time. Unlike other central nervous system regions, in which Ptf1a plays a specific role in the generation of inhibitory GABAergic cell types, in the retina, both GABAergic (horizontal and some subtypes of amacrine cells, including the displaced types) and glycinergic cells (some amacrine cell subtypes) arise from Ptf1a:GFP cells, suggesting that the different neurotransmitter identity of these inhibitory neurons is determined by other factors, which have been suggested to act much earlier in progenitor cells [39].

Among mammalian species in which amacrine cells have been classified to date, the estimated number of subtypes of amacrine cells ranges between 25 and 30 [30,32,37], while in the roach, a related teleost fish, they have been suggested to consist of as many as 43 major types [40]. Here we show that a similar variety of amacrine cell subtypes had already developed by 120 hpf. Anatomical features (for example, lamination, remodelling and synapses) are present and the visual system is functional by 3 days post-fertilization [29,33-36], and immunohistochemical subtype markers already label distinct subpopulations at this time. Indeed, our morphological classification scheme identified 28 different subtypes, comparable to the variety encountered in mammals [30,32,37]. The stratification of cell types identified in this study was relatively simple (many monostratified types and simple branching patterns compared to, for example, fountain and recurving and AII cells) compared to those described in mammals and might reflect the relatively simple visual circuitry of the embryonic zebrafish retina.

Other trends described in different species also hold true for the zebrafish classification scheme presented here. Firstly, wide-field amacrine cells tend to be monostratified whilst narrow-field amacrine cell types often stratify more broadly [37], and may form a modulating link between different synaptic circuits analysing the same visual area in space. However, direct homologies cannot be drawn between most of the zebrafish amacrine cell types described here and the described mammalian amacrine cell subtypes. This does not come as a surprise, given that some of the other classified cell types, such as the bipolar cells, also differ vastly in their morphologies in zebrafish compared to mammalian retinas [18,24,41-45]. Secondly, the three displaced amacrine cell types described here all have neurites that stratify within the inner half of the IPL, which is also commonly observed in displaced amacrine cells of other species [31,37,46-48]. In mammals, narrow-field amacrine cells tend to be glycinergic and larger amacrine cells tend to be GABAergic, but this trend does not hold true for other vertebrates such as fish (reviewed by [32]). Whilst we could not determine the main neurotransmitter identity for specific types of amacrine cells, displaced amacrine cells in zebrafish seem to be GABAergic (since antibodies to GABA labelled somas in the GCL, whereas antibodies to glycine did not), as previously described in different vertebrates [49-55]. Interestingly, the three types of zebrafish displaced amacrine cells (GABAergic) described here were all of narrow-field morphology, suggesting a difference between the neurotransmitter identity of narrow versus wide-field amacrine cells in different species. Lastly, many of the smaller types occurred at a higher frequency than the types with larger neurite arbors, which is consistent with the inherent idea that cells of a given subtype must be able to cover the entire retina (thus, cells with smaller arbors occur at a higher density and would be found more frequent if labelled randomly) in order to carry out their specific function across the visual field [56].

Similar morphologies of each of the seven amacrine cell types (previously reported in adult zebrafish [18]) were found in this sample (Table 1). One previously described type, Aon-4, matches one of our unclassified cells (narrow-field smooth S3 to S5, Figure 9); this was only encountered once in this sample set, suggesting that cells with this morphology might indeed be a true subtype. However, to be conservative, we did not feel confident to make a class with features only found in one example, in case it represents a rare developmental abnormality. Whilst some types had only two examples, it is far less likely that different cells had developmental errors leading to the same morphology. Secondly, all of the types for which only two examples were encountered were of the medium- or wide-field morphology and some had processes spanning a quarter of the retina, such that a low frequency might relate to a lower density required for these types as discussed above. Indeed, some mammalian subtypes were encountered at similarly low frequencies [57], so such low occurrences come as no large surprise.

The cell types that are matched morphologically between the current and the previous study [18] are much larger in the adult zebrafish, but the main features, such as stratification and relative width (compared to IPL size, for example), match well. This suggests that eye growth is achieved by not only addition of new cells from the ciliary margin, but also growth of individual cells. The bistratified Aon-s1/s5 described by Connaughton and colleagues [18] does not seem to have a large neurite arbor, as our equivalent type did in the embryonic retina, indicating that some cell types might grow more in size than others. The descriptive summary of a total of 27 adult zebrafish amacrine cells presented in an abstract by the same group [58] also matches the types described here.

Some immunohistochemical subtype-specific markers label types with somas located at a particular depth of the INL [59], suggesting that somal location might be a subtype-specific criterion. In some classified types the somas were consistently located in the same INL layer (for example, nearest to the IPL or furthest away from the IPL adjacent to the bipolar somas), with wide-field cells having somas nearest to the bipolar somas. Other types had randomly placed somas, which could mean that different groups of amacrine cells were combined together or that somal positioning is somewhat random in these subtypes (perhaps related to the order of development) and may not carry subtype-specific significance. Not surprisingly, single or combinations of cell types classified in this study could account for the immunohistochemical staining pattern obtained by the vast majority of amacrine-specific antibodies found by Yazulla and Studholme [59].

The main point of the classification at this age is to reveal the diversity of amacrine cell types within the embryonic zebrafish retina that can be used as a basis for investigating how different functional manipulations of genes and/or external signals affect the different cell types already present at this early age. Subtypes that differ in very subtle features might have been erroneously grouped together here; however, the interesting finding is that by analysing relatively simple unambiguous features (mainly stratification depth and width), we can already distinguish dozens of different types. A more detailed description of the morphology of individual amacrine cell types (such as branching patterns or density, degree of ellipticity of neurite arbor) cannot be carried out with this marker, due to the decrease of stable GFP protein by 120 hpf (as the RNA expression ceases by 65 hpf). By then, the signal strength was not sufficiently bright to perform more extensive three-dimensional reconstructions as has been done in other classification studies [60].

## Conclusion

Ptf1a is expressed transiently in postmitotic precursors that differentiate into all the inhibitory cells in the zebrafish retina, suggesting that this aspect of fate determination might occur in a critical temporal window during the early development of individual neurons that then differentiate into different horizontal and amacrine cell subtypes. Cell type identity can be determined by soma location, stratification of processes and staining patterns with immunohistochemical markers, which reveals the presence of distinct subtypes. It will be interesting in future to examine if and how Ptf1a interacts with other factors to determine the identity of these subtypes and whether these subtypes come from lineage restricted progenitors such that certain subtypes of inhibitory cells (that is, glycinergic versus GABAergic) tend to have clonal relationships with specific excitatory cell types in the retina. We hope our classification schemes at this early developmental stage of the zebrafish will aid future studies in discovering other genes that influence specific subtypes of amacrine cells by, for example, comparing the different subtypes present in normal and mutant animals.

## Materials and methods

### Animals

Zebrafish were maintained and bred at 26.5°C. Embryos were raised at 28.5°C and staged as previously described [61] in hours post-fertilization. Embryos were treated with 0.003% phenylthiourea (Sigma, Gillingham, Dorset, UK)) starting at 11 to 24 hpf to delay pigment formation in the eye. All procedures were carried out under the project licence PL80/2198 approved by the UK Home office and by the Local Ethical Review Panel at the University of Cambridge.

A transgenic line expressing GFP under the control of the *ptf1a *promoter region Tg(*ptf1a*:*GFP*) was generated using bacterial artificial chromosome recombineering [29] and kindly provided to us by Steven D Leach (John Hopkins Medical Institutions, Baltimore, USA).

### Construct injections

For mosaic expression of fluorescent proteins and morphological characterization of single cells, embryos were injected with *ptf1a:GFP *bacterial artificial chromosome (kind gift of Steven D Leach) constructs (1 nl of 30 ng/μl) into the cell at the one-cell stage. To identify host cells during transplantations, *H2B:RFP *mRNA (1 nl of 100 to 150 ng/μl) transcribed from a pCS2-*H2B:RFP *(with SP6 RNA polymerase using the mMessage mMachine Kit (Ambion, Applied Biosystems, Warrington, UK)) construct was injected into the yolk at the one-cell stage. DNA and RNA injections were made using a micromanipulator-mounted micropipette and a Picospritzer microinjector.

### Blastomere transplantation

To follow single progenitors and their progeny, 10 to 50 cells from blastomeres were transplanted from labelled embryos (GFP transgenes previously injected with *H2B:RFP *mRNA to obtain a general nuclei labelling) into the animal poles of unlabeled blastulas. In brief, embryos were embedded in a drop of 2% methylcellulose on a coverslip, and cells were transferred from one donor to up to four hosts with a glass micropipette as described previously [62].

### Immunohistochemistry

#### Antibodies

The primary antibodies used in this study are listed in Table 2. Application dilutions, source details, immunogens used in the generation of these antibodies and the tests used to verify their specificity are provided. Secondary antibodies used were goat or donkey anti-mouse, anti-rabbit or anti-goat IgG conjugated to Alexa 488, 594 or 647 fluorophores (1:1,000 to 1:2,000 dilution; Molecular Probes, Eugene, OR, USA).

**Table 2:** Primary antibody information

**Antibody**	**Dilution**	**Source**	**Immunogen**	**Specificity**
rb anti-calretinin	1:2000	Millipore, AB5054	Recombinant rat calretinin	Immunoblot (manufacturer)
rb anti-GABA	1:500	Sigma, A2052	GABA-BSA	Dot-blot analysis (manufacturer)
ms anti-GFP	1:500	Roche, 11814460001	Partially purified recombinant *Aequorea victoria *GFP	Western blot and immunoprecipitation (manufacturer)
rb anti-glutamine synthetase	1:50	Millipore, MAB302 clone GS-6	Glutamine synthetase purified from sheep brain	Western blot of sheep and rat brain (manufacturer)
rb anti-glycine	1:200	Millipore, AB139	Glycine-glutaraldehyde-BSA	Free floating PAP technique on rat spinal cord (manufacturer)
rb anti-Hermes	1:400	Associate Professor Malgorzata Kloc., University of Texas, USA	Recombinant His-tagged Hermes protein	Western blot to recognise my-tagged Hermes fusion protein [66]
rb anti-PKCβ1	1:150	Santa-Cruz Biotechnology, (C-16) sc-209	Carboxyl terminus of PKC β1 of human origin	Western blot in NIH/3t3, 3611-RF, A-431, HeLA and Jurkat whole cell lysate (manufacturer)
rb anti-prox1	1:200	Millipore, MAB5652 clone 5G10	Recombinant human Prox1 protein	Western blot in fetal mouse brain lysate (manufacturer)
ms anti-parvalbumin	1:1000	Millipore, MAB1572 clone parv19	Frog muscle parvalbumin directed against epitope at first Ca^2+ ^binding site	Western blot (manufacturer)
rb anti-sox2	1:200	Millipore, MAB5603	Synthetic peptide corresponding to human Sox2	Western blot of whole cell or nuclear extracts (manufacturer)
ms anti-5E11	1:50	Associate Professor James M Fadool, Florida State University, USA	Whole zebrafish retina (antibody was subsequently purified)	Western blot of total retina protein [67]
ms anti-Zpr1	1:500	ZIRC, Monoclonal Antibody Facility, University of Oregon	Adult zebrafish protein	Clones selected on basis of staining pattern
ms anti-Zpr3	1:400	ZIRC, Monoclonal Antibody Facility, University of Oregon	Adult zebrafish protein	Clones selected on basis of staining pattern

BSA, bovine serum albumin; GABA, γ-aminobutyric acid; ms, mouse; PAP, peroxidase anti peroxidase; PKC, protein kinase C; rb, rabbit; ZIRC, Zebrafish International Resource Center.

For most antibodies, wholemount zebrafish embryos were fixed with 4% paraformaldehyde (PFA) in 0.1 M phosphate buffer, pH7.4 overnight at 4°C, rinsed, cryoprotected in 30% sucrose in 0.1 M phosphate buffer, embedded in OCT and cryosectioned at 14 μm thickness. For GABA and glycine immunohistochemistry, embryos were anaesthetized with 0.4 mg/ml MS222 and aligned on a slide. The head was cut off using a razor blade (allowing better penetration) and immediately immersion fixed in 2% PFA/2% glutaraldehyde in 0.1 M phosphate buffer for 2 days at 4°C. These embryos were embedded in LRW resin as described previously [63]. Sections were cut at 0.5 to 1 μm thickness on an Ultracut E microtome (Reichert Microscope Service, Depew, NY, USA) and dried on a heatblock.

All immunohistochemistry staining steps were performed at room temperature unless stated otherwise. For Sox2 immunohistochemistry, antigen retrieval was performed by immersing sections in 0.01 M sodium citrate buffer, pH6.0 at 95°C for 10 minutes prior to the blocking step. For GABA and glycine immunohistochemistry, sections were treated with 0.1% sodium borohydride (NaBH_4_) in 0.2% triton-X in phosphate-buffered saline (PBS) for 10 minutes.

For immunolabelling, sections were incubated in blocking buffer (10% heat-inactivated goat serum, 1% bovine serum albumin, 0.2% Triton X-100 in PBS for cryostat sections and 10% heat-inactivated goat serum, 0.1% Tween-20, 0.5% Triton X-100 in PBS for plastic sections) for 30 minutes. Sections were incubated in primary antibodies diluted in the blocking buffer overnight. After rinsing with PBS, sections were incubated in secondary antibodies diluted in the blocking buffer for 2 hours. Antibodies were rinsed off with PBS and 4',6-diamidino-2-phenylindole (DAPI; 1:1,000) was added to the last rinse. Sections were coverslipped with Fluorosave mounting medium (Calbiochem, Merck Chemicals Ltd, Nottingham, UK).

### Whole mount *in situ *hybridization

*In situ *RNA hybridization was performed as previously described [5]. The *ptf1a *zebrafish gene (NM 207641) obtained in a pcDNA 3.1 Invitrogen plasmid (kind gift from Steven D Leach) was subcloned into the pCS2+ vector (*Eco*R1 sites). After sequencing to confirm the gene sequence, antisense (cut with *Bam*H1, transcribed with T7) and sense (cut with *Xho*I, transcribed with SP6) control digoxigenin-labelled riboprobes were synthesized from this construct.

Embryos underwent a stepwise dehydration series into 100% methanol and subsequent rehydration into 0.1% Tween in PBS. Permeabilization was achieved using age-dependent concentrations of proteinase K treatment for 25 minutes at room temperature, followed by post-fixation in 4% PFA in PBS. After prehybridization, riboprobe hybridization was performed at 68°C for 20 to 65 hours. Hybridized probes were detected with nitroblue tetrazolium chloride/5-bromo-4-chloro-3-indolyl-phosphate, toluidine salt (NBT/BCIP BM Purple, Roche Products Ltd, Welwyn Garden City, UK).

The GFP signal in transgenic lines was recovered using a mouse anti-GFP primary antibody and an anti-mouse secondary IgG antibody conjugated to the Alexa 488 fluorophore. Some embryos were subsequently post-fixed in 4% PFA in PBS, embedded in OCT, cryostat sectioned at 40 μm thickness and coverslipped with fluorosave mounting medium.

### Imaging of fixed samples

Whole or partial embryo images were acquired on a dissecting stereo microscope equipped with epi-fluorescence (Leica MZ FLIII). Photomicrography of wholemount eyes or sections was performed with either a laser confocal system (Leica TCS-NT confocal laser scanning microscope using a Leica 40×, 1.2 NA or Leica 63×, 1.2 NA water immersion objective) or with Nikon fluorescence microscopes, equipped with cooled charge-coupled device (CCD) Hamamatsu Orca cameras and automated *z*-drive and fluorescence shutters. At the confocal microscope a 405 nm laser line was used to visualise DAPI, a 488 nm argon laser line was used to visualise GFP and Alexa 488 fluorophore, a 594 nm laser line was used for Alexa 594 fluorophore excitation and a 633 nm laser line was used to visualise Alexa 647 fluorophore. Emission was detected using individual descanned PMT detectors. Optical sections of 1 μm thickness were taken through a volume of the retina up to 100 μm in depth and Kalmann averaged two or four times. Image data were acquired and stored as TIFF files using Leica TCS NT or Leica LCS software. Noise removal on stacks was performed using Volocity (Improvision, Coventry, UK) and brightness and contrast of images was adjusted using Adobe Photoshop.

### Live imaging of wholemounted embryos

Embryo processing and four-dimensional imaging were performed as described previously [64]. Usually, stacks about 40 μm thick, composed of optical sections separated by 1 μm, were taken every 6 to 10 minutes during an average period of 16 to 28 hours to study the developmental onset of Ptf1a:GFP expression and every 10 or 30 minutes at 120 hpf for up to 24 hours to study the stability of amacrine subtype morphology at this embryonic stage. Laser power was kept at a minimum to avoid bleaching and phototoxicity. The four-dimensional data thus obtained were processed and analyzed with Volocity.

### Analysis

Subtypes of amacrine cells were classified using morphological features. As immunohistochemical markers for subtypes or groups of subtypes of amacrine cells seem to label somas within a particular layer of the INL, the position of amacrine cells was recorded. The amacrine layer (inner half of the INL) usually contains three to four layers of somas; thus, individual cells were assigned as having somas closest to the IPL, closest to the bipolar half of the INL or in the in-between layer. The zebrafish retina also contains displaced amacrine cells with somas in the ganglion cell layer and these always had their somas in the layer immediately adjacent to the IPL.

Measurements were performed using the ImageJ [65] line length measurement tool by focusing back and forth through the stacks of images. The minor and major diameters of somas were measured. The most distinguishing feature among groups is the morphology and stratification pattern of the neurites.

Cells were included only if they were completely separable from neighbouring cells in three-dimensional space. Some cells would seem intermingled in projections of stacks, but were isolated in focal depth and could be unambiguously separated in the individual images, which were used during analysis. The stratification depth (outermost and innermost neurite) was measured as a percentage of IPL depth (0% being the border with the INL, 100% being the border with the GCL) with at least two measurements taken at the extreme ends for wide-field amacrine cells. For classification and descriptive purposes the IPL was subdivided into six equal sublaminas (S1 to S3 represent the outer/OFF half and S4 to S6 represent the inner/ON half of the IPL) as used previously in zebrafish retinal neuron classification [18]. Cells with processes contained within a single sublamina were classified as monostratified, others as multistratified confined either to the OFF sublamina (S1 to S3), ON sublamina (S4 to S5) or both.

Amacrine cells have been classically subdivided into narrow-field, medium-field or wide-field classes, based on the extent of their neurites. Thus, the width of the neurites was measured and cells were classified accordingly. The process of choosing the cutoff size between groups is explained in the results section.

Lastly, morphological features of the neurites were used as a final classification parameter. Most notably, some cells had smooth processes, whereas others had large varicosities and some had arbors with very characteristic shapes.

## Abbreviations

DAPI: 4',6-diamidino-2-phenylindole; GABA: γ-aminobutyric acid; GCL: ganglion cell layer; GFP: green fluorescent protein; hpf: hours post-fertilization; INL: inner nuclear layer; IPL: inner plexiform layer; PBS: phosphate-buffered saline; PFA: paraformaldehyde; PKC: protein kinase C; Ptf1a: pancreas transcription factor 1a; RFP: red fluorescent protein.

## Competing interests

The authors declare that they have no competing interests.

## Authors' contributions

PRJ carried out the experiments and wrote the initial manuscript. WAH conceived of the study, supervised the work, which was done in his laboratory, and revised the manuscript.

## Supplementary Material

Additional file 1 **Onset of Ptf1a expression in individual cells**. Time-lapse (10 minutes/frame) of wild-type hosts with transplanted cells from Tg(*ptf1a:GFP)/H2B:RFP *RNA injected embryo to reveal individual cells (38 to 58 hpf). Transplanted (red) cells turn on Ptf1a:GFP expression in the apical half (top) of the developing neuroepithelium, but not always at the apical surface, as cells have started migrating basally. The cells in this movie can be followed through the movie and are shown to move to the developing amacrine layer in the centre of the retina, without ever dividing again (Figure 4). Framerate: 0.1 seconds/frame. Click here for file

Additional file 2 **Morphology of example horizontal cells in Ptf1a:GFP DNA injected embryos (120 hpf)**. Micrographs showing single images or extended focus views. The nuclear stain DAPI was used to reveal the retinal layers. Horizontal cells have somas in the outer inner nuclear layer immediately adjacent to the outer plexiform layer. Different horizontal cell types have more or less elongated somas and the dendritic trees of different types can extend to form relatively smaller (for example, (A, B, C)) or larger (for example, (E, H)) arbors. Pattern of dendritic tips could not be distinguished in this vertical view and horizontal cells were not further classified, although based on the morphology shown in this vertical view, examples of the previously described types of horizontal cells could be found. Some well-labelled cells also had a distinct axon (G). Scale bar = 20 μm. Click here for file

Additional file 3 **Morphology of additional types of amacrine cells in Ptf1a:GFP DNA-injected embryos (120 hpf)**. Micrographs show single images only and some of the joining neurites and/or somas are thus not in focus in the shown images. The nuclear stain DAPI was used to reveal the retinal layers. As described in Figure 7, different subtypes can be distinguished by the stratification depth, breadth, neurite arbor width and smooth or beaded neurite morphology. **(A-H, K)** Narrow-field amacrine cell types. **(I, J) **Medium-field amacrine cell types. **(K-N) **Wide-field amacrine cell types. dAC, displaced amacrine cell; IPL, inner plexiform layer; MF, medium-field; mono, monostratified; NF, narrow-field; WF, wide-field. Scale bar = 20 μm. Click here for file
