# Supplementary figures and images for: Ptf1a is expressed transiently in all types of amacrine cells in the embryonic zebrafish retina
Source: Neural Dev. 2009 Sep 4;4:34. doi: 10.1186/1749-8104-4-34 (PMC2746205; doi:10.1186/1749-8104-4-34)

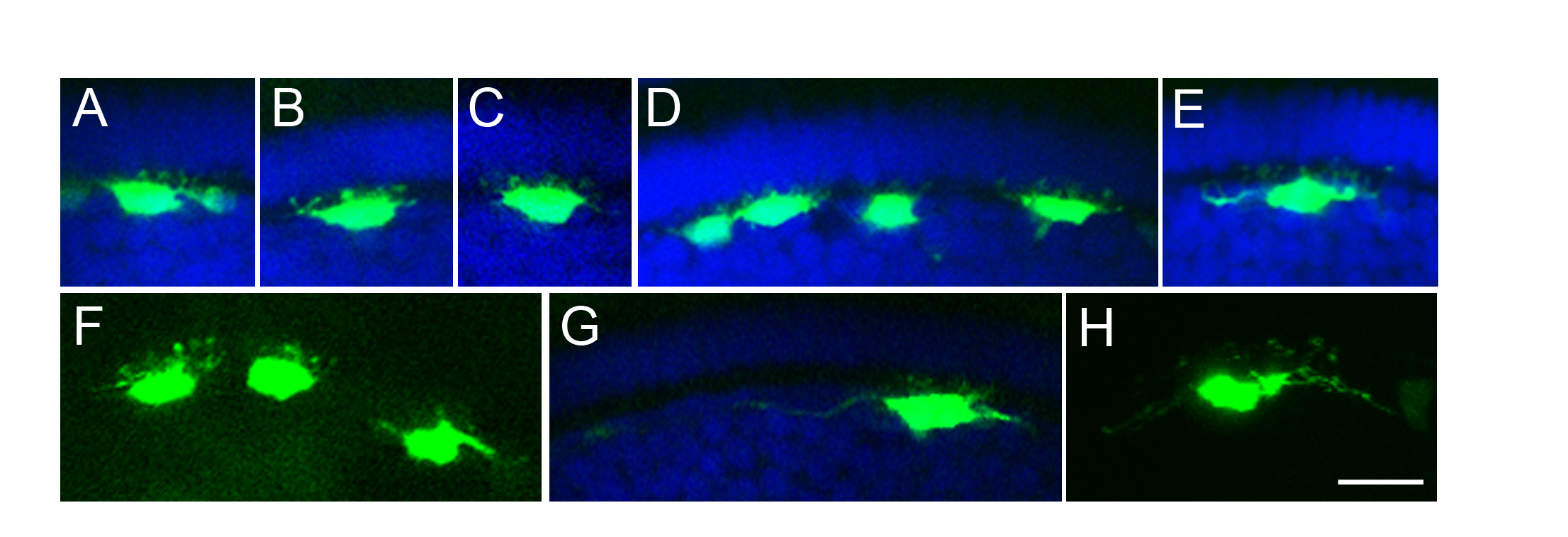

Supplement: Additional file 2 — Morphology of example horizontal cells in Ptf1a:GFP DNA injected embryos (120 hpf). Micrographs showing single images or extended focus views. The nuclear stain DAPI was used to reveal the retinal layers. Horizontal cells have somas in the outer inner nuclear layer immediately adjacent to the outer plexiform layer. Different horizontal cell types have more or less elongated somas and the dendritic trees of different types can extend to form relatively smaller (for example, (A, B, C)) or larger (for example, (E, H)) arbors. Pattern of dendritic tips could not be distinguished in this vertical view and horizontal cells were not further classified, although based on the morphology shown in this vertical view, examples of the previously described types of horizontal cells could be found. Some well-labelled cells also had a distinct axon (G). Scale bar = 20 μm. [file 1749-8104-4-34-S2.tiff]

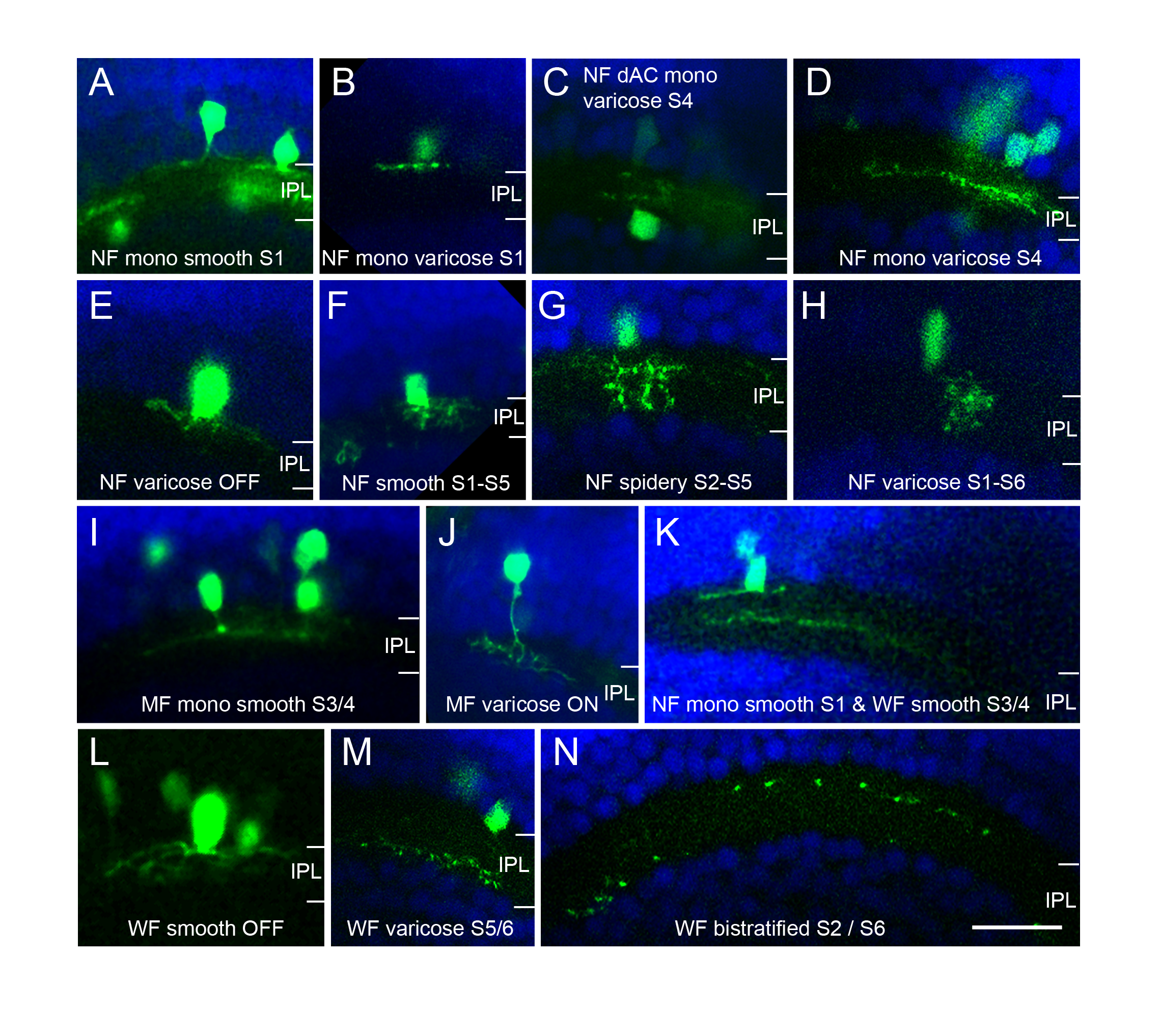

Supplement: Additional file 3 — Morphology of additional types of amacrine cells in Ptf1a:GFP DNA-injected embryos (120 hpf). Micrographs show single images only and some of the joining neurites and/or somas are thus not in focus in the shown images. The nuclear stain DAPI was used to reveal the retinal layers. As described in Figure 7, different subtypes can be distinguished by the stratification depth, breadth, neurite arbor width and smooth or beaded neurite morphology. (A-H, K) Narrow-field amacrine cell types. (I, J) Medium-field amacrine cell types. (K-N) Wide-field amacrine cell types. dAC, displaced amacrine cell; IPL, inner plexiform layer; MF, medium-field; mono, monostratified; NF, narrow-field; WF, wide-field. Scale bar = 20 μm. [file 1749-8104-4-34-S3.tiff]
